# Long noncoding RNA Glis2 regulates podocyte mitochondrial dysfunction and apoptosis in diabetic nephropathy via sponging miR‐328‐5p

**DOI:** 10.1111/jcmm.18204

**Published:** 2024-03-20

**Authors:** Ting Wang, Yanxia Chen, Zhihong Liu, Jing Zhou, Na Li, Yue Shan, Yinxi He

**Affiliations:** ^1^ Department of Endocrinology Second Hospital of Hebei Medical University Shijiazhuang Hebei P.R. China; ^2^ Department of Orthopaedic Trauma The Third Hospital of Shijiazhuang Shijiazhuang Hebei P.R. China

**Keywords:** competing endogenous RNA (ceRNA), diabetic nephropathy (DN), long noncoding RNAs (lncRNAs), mitochondrial dysfunction, podocyte apoptosis

## Abstract

Podocyte apoptosis exerts a crucial role in the pathogenesis of DN. Recently, long noncoding RNAs (lncRNAs) have been gradually identified to be functional in a variety of different mechanisms associated with podocyte apoptosis. This study aimed to investigate whether lncRNA Glis2 could regulate podocyte apoptosis in DN and uncover the underlying mechanism. The apoptosis rate was detected by flow cytometry. Mitochondrial membrane potential (ΔΨM) was measured using JC‐1 staining. Mitochondrial morphology was detected by MitoTracker Deep Red staining. Then, the histopathological and ultrastructure changes of renal tissues in diabetic mice were observed using periodic acid‐Schiff (PAS) staining and transmission electron microscopy. We found that lncRNA Glis2 was significantly downregulated in high‐glucose cultured podocytes and renal tissues of db/db mice. LncRNA Glis2 overexpression was found to alleviate podocyte mitochondrial dysfunction and apoptosis. The direct interaction between lncRNA Glis2 and miR‐328‐5p was confirmed by dual luciferase reporter assay. Furthermore, lncRNA Glis2 overexpression alleviated podocyte apoptosis in diabetic mice. Taken together, this study demonstrated that lncRNA Glis2, acting as a competing endogenous RNA (ceRNA) of miRNA‐328‐5p, regulated Sirt1‐mediated mitochondrial dysfunction and podocyte apoptosis in DN.

## 1 INTRODUCTION

Diabetic nephropathy (DN) is one of the most serious microvascular complications of diabetes and the leading cause of chronic kidney disease (CKD) and end‐stage renal disease in the world.^1^ Clinically, DN is characterized by progressive proteinuria with a subsequent glomerular filtration rate reduction, resulting in irreversible renal dysfunction and uremic symptoms. The histopathological features of DN involve several complicated mechanisms, including glomerular hypertrophy, thickening of the glomerular basement membrane, massive accumulation of the extracellular matrix, podocyte reduction, and ultimately the development of interstitial fibrosis and glomerular sclerosis. Podocytes, which are located outside the glomerular capillaries, are a key component of the kidney filtration barrier. When podocytes suffer from various external stimuli including high glucose, they undergo foot process effacement and loss of slit diaphragms, leading to podocyte apoptosis.^2^ Podocyte apoptosis could disrupt the glomerular filtration barrier and induce the appearance of albuminuria, resulting in progressive loss of kidney function,^3^ which has been widely considered as a crucial pathological mechanism in the progress of DN. Thus, exploring the therapies for preventing podocyte apoptosis or promoting podocyte repair may help to seek efficient targets and prospective strategies for DN.

Long noncoding RNAs (lncRNAs) are a class of RNA transcripts with no protein‐coding ability longer than 200 nucleotides in length.^4^ More and more research related to lncRNAs has been carried out extensively in recent years, leading to the discovery of their significant roles in gene modulation through epigenetic, transcriptional and post‐transcriptional regulatory mechanisms.^5^ Growing evidence has indicated that lncRNAs play vital roles in various physiological and pathological processes of human diseases.^6^, ^7^ LncRNAs also participated in the progression of DN, including podocyte apoptosis.^8^

To further investigate the lncRNA expression profiles and the role of unidentified lncRNAs in podocyte apoptosis, high‐throughput RNA‐sequencing (RNA‐seq) technologies and bioinformatics analytical were used to examine differentially expressed lncRNAs in normal‐glucose and high‐glucose cultured mouse podocytes. Among these lncRNAs, ENSMUST00000122896 (lncRNA Glis2 for short) was a novel lncRNA and was chosen for further research. The function of unidentified lncRNA Glis2 and its molecular mechanism in podocyte apoptosis has not been reported until now. The present study aimed to investigate the function and underlying molecular mechanism of unidentified lncRNA Glis2 in podocyte apoptosis, maybe providing a novel therapeutic target against DN.

## 2 MATERIALS AND METHODS

### 2.1 Cell culture

The conditionally immortalized mouse podocytes were purchased from the Cell Culture Center (PUMC, CAMS, Beijing, China). The podocytes were cultured in Dulbecco's modified eagle medium (DMEM) with 10% fetal bovine serum, 100 U/mL penicillin and 100 μg/mL streptomycin at 37°C under a 5% CO_2_ atmosphere incubator. Podocytes were subsequently sub‐cultured at 80%–90% confluence for further experiments. The culture medium was changed every day or 2 days. To mimic a high‐glucose environment or normal growth status, the podocytes were incubated in DMEM with high glucose (HG, 25 mmol/L glucose) or normal glucose (NG, 5.5 mmol/L glucose plus 19.5 mmol/L mannitol), respectively. Mannitol was added to the cell incubation medium to ensure osmolar equivalence in the NG group.

### 2.2 RNA sequencing (RNA‐Seq)

Total RNA from the podocytes of the NG and HG groups were extracted using TRIzol® Reagent (Life Technologies) in accordance with the manufacturer's instructions. RNA concentration was measured using Qubit® RNA Assay Kit in Qubit®2.0 Fluorometer (Life Technologies, CA, USA). The total RNA was purified by depleting ribosomal RNA using the Ribo‐off rRNA Depletion kit (Vazyme Biotech Co., Ltd, Nanjing, China). Then, these samples were used for constructing a cDNA library using VAHTS™ Stranded mRNA‐seq Library Prep Kit for Illumina® (Vazyme Biotech Co., Ltd, Nanjing, China). cDNA libraries were sequenced using an Illumina Novaseq6000 sequencer (Illumina Inc., San Diego, CA, USA), which was conducted by Sangon Biotech (Shanghai, China). Differentially expressed lncRNAs with statistical significance between NG and HG groups were identified through *p*‐value/false discovery rate (FDR) filtering. A volcano plot filtering approach (|log2(fold change)| ≥ 1.0; *q* value ≤0.05) was considered as differentially expressed lncRNAs between NG and HG groups.

### 2.3 Cell transfection

For transfection, the podocytes from each group were seeded in six wells and incubated for 24 h. After the cell fusion degree was approximately 60%–80%, the transfection was conducted using opti‐MEM and lipofectamine 2000 reagents (Invitrogen, Carlsbad, CA, USA) according to the manufacturer's protocol. To overexpress lncRNA Glis2, pcDNA3.1‐lncRNA Glis2 was constructed by inserting lncRNA Glis2 coding sequence into plasmid pcDNA3.1, and pcDNA3.1 empty vector was used as a negative control. For lncRNA Glis2 knockdown, small interference RNA targeting lncRNA Glis2 (siRNA‐lncRNA Glis2) was synthesized by Hunan Fenghui Biotechnology (Changsha, China). siRNA‐NC was used as a negative control. For miR‐328‐5p overexpression and knockdown, miR‐328‐5p mimics, miR‐328‐5p inhibitors and a matched miRNA negative control were synthesized and purified by Hunan Fenghui Biotechnology (Changsha, China), respectively.

### 2.4 Animal experiments

All experimental procedures were in accordance with the institutional animal care and use committee and approved by the Animal Care and Ethics Committee of the Second Hospital of Hebei Medical University (approval ID: 2022‐AE051). Eight‐week‐old male C57BLKS/J db/db mice and their normal littermates (db/m) were purchased from SPF (Beijing) Biotechnology Co., Ltd. (Beijing, China). All mice were fed in a standard animal house with a 12‐h light/12‐h dark cycle and given ad libitum access to food and water. After 1 week of adaptive feeding, db/db mice were randomly divided into three groups: db/db group, db/db + pcDNA3.1‐NC group (db/db mice injected with empty vector) and db/db + pcDNA3.1‐lncRNA Glis2 group (db/db mice injected with 50 μL 1 × 10^6^ infective units lncRNA Glis2 overexpression lentivirus every week). Meanwhile, db/m mice served as a control group. At the end of 12 weeks after treatment, mice were kept in individual metabolic cages for urine collection to measure the urinary albumin‐to‐creatinine ratio (UACR). Blood samples were obtained for blood glucose, blood urea nitrogen (BUN), and serum creatinine (Scr). Then, the mice were sacrificed and the kidneys were harvested immediately for follow‐up experiments.

### 2.5 Flow cytometry

After 48 h transfection, cell apoptosis from each group was detected with an Annexin V‐FITC and propidium iodide (PI) double staining kit (MultiSciences Biotechnology Corporate Limited, China) by flow cytometry (BD Biosciences, USA). Cells were collected and single‐cell suspensions (1 × 10^6^ cells/mL) were prepared. Then the cells were resuspended in a binding buffer. After that, the cells were dual stained with annexin‐V FITC/propidium iodide (PI) at room temperature for 5 min. Finally, apoptotic cells were detected by flow cytometry.

### 2.6 Measurement of cell viability

Cell viability was assessed with a Cell Counting Kit‐8 (CCK‐8) assay. The podocytes were seeded in a 96‐well plate and cultured overnight at 37°C. After that, each well was mixed with 10 μL CCK‐8 agent (Sigma‐Aldrich, St Louis MO, USA). After 1 h incubation, the light absorbance value was measured using a microplate reader at 450 nm.

### 2.7 Immunofluorescence

For immunofluorescence, the coverslips were fixed in 4% paraformaldehyde and permeabilized with 0.5% Triton X‐100. Next, the coverslips were blocked in 5% bovine serum albumin for 1 h. Subsequently, the coverslips were incubated with the following primary antibodies (anti‐podocin; anti‐nephrin) at 37°C overnight. The coverslips were washed and incubated with the corresponding secondary antibodies at room temperature for 1 h. After being tinted with DAPI, the images were observed with a fluorescent microscope (Olympus FV10‐ASW, Tokyo, Japan).

### 2.8 Quantitative real‐time polymerase chain reaction (qRT‐PCR)

Total RNA from cultured cells or renal tissues was isolated using TRIzol reagent following the manufacturer's instructions. The quality and concentration of RNA were detected using a NanoDrop 2000 spectrophotometer (Thermo Fisher Scientific, Waltham, MA, USA). The RNA quality was examined based on the absorbance ratio at 280/260 nm, and the RNA concentration was examined based on absorbance at 260 nm. A reverse transcription experiment was performed by the Revert Aid First Strand cDNA Synthesis kit (Thermo Fisher Scientific). Then, qRT‐PCR was performed using SYBR®Premix Ex Taq™ (Takara, Dalian, China). The relative quantitative result was calculated using the 2^−△△Ct^ method. The expression of GAPDH or U6 was set as the internal normalization control for lncRNA or miRNA.

### 2.9 Fluorescence in situ hybridization (FISH)

The subcellular distribution of lncRNA Glis2 was detected using a FISH kit (Boster Biological Technology Co. Ltd, Wuhan, China) according to the manufacturer's instructions. The lncRNA Glis2 FISH probe was designed and synthesized by Servicebio Technology (Wuhan, China). The slides were fixed in 4% paraformaldehyde at room temperature for 30 min. Next, the cells were permeabilized with cold PBS containing 0.5% Triton X‐100. Subsequently, the cells were incubated with the fluorescence probe at 37°C overnight. Then, the slides were stained with DAPI. The images were visualized with a fluorescent microscope (Olympus FV10‐ASW, Tokyo, Japan).

### 2.10 Dual luciferase reporter assay

The dual luciferase reporter assay was constructed to determine the direct interaction of lncRNA Glis2 and miR‐328‐5p, as well as miR‐328‐5p and Sirtuin1 (Sirt1). To construct plasmids used in dual luciferase reporter assay, the predicted 3′‐UTR sequence of lncRNA Glis2 (or Sirt1) interacting with miR‐328‐5p and an artificially mutated sequences within the predicted target sites were synthesized and inserted into pmirGLO vector (Cosmo Bio, Tianjin, China), respectively. Then, podocytes were co‐transfected with the wide type (wt) or mutated (mut) luciferase reporter plasmid and miR‐328‐5p mimics or NC mimics using lipofectamine 2000 reagents (Invitrogen, Carlsbad, CA, USA) according to the manufacturer's instructions. After 48 h of transfection, luciferase activity was detected using a dual luciferase assay system (Promega, Madison, WI, USA). The relative luciferase activity was normalized to Renilla luciferase activity.

### 2.11 Mitochondrial membrane potential (ΔΨM)

The ΔΨM of podocytes was detected using mitochondrial membrane potential assay kit with JC‐1 (Beyotime Biotechnology, Shanghai, China) in accordance with the manufacturer's instructions. The cultured podocytes were washed with PBS and then incubated with JC‐1 working solution for 30 min at 37°C in darkness. The podocytes were rinsed with JC‐1 staining buffer. After that, the images were observed with a fluorescent microscope (Olympus FV10‐ASW, Tokyo, Japan). The values were expressed as the ratio of red relative to green fluorescence levels.

### 2.12 Mitochondrial morphology

The mitochondrial morphology was observed by MitoTracker™ Deep Red FM staining. The cultured podocytes were incubated with MitoTracker Deep Red (Yeasen Biotechnology, Shanghai, China) for 30 min at 37°C in the dark. Fluorescence images were obtained using a fluorescent microscope (Olympus FV10‐ASW, Tokyo, Japan).

### 2.13 Adenosine triphosphate (ATP)

ATP levels were assessed using an ATP Assay Kit (Beyotime Biotechnology, China). Briefly, the cultured cells were harvested and lysed with lysis reagent and then centrifuged at 12,000 × *g* for 10 min. The supernatant was collected and mixed with ATP detection solution in a 96‐well plate afterwards. The light absorbance value was detected using a microplate reader.

### 2.14 Western blot

Total protein from cultured podocytes or renal tissues was extracted using RIPA lysis buffer. The protein concentration was detected by Bradford protein content assay kit (Boster Biological Technology Co. Ltd, Wuhan, China) following its standard protocol. An equal amount of protein was separated by 10% sodium dodecyl sulfate‐polyacrylamide gel electrophoresis (SDS‐PAGE) and transferred onto a polyvinylidene difluoride (PVDF) membrane. Then, the membranes were blocked with 5% skim milk or bovine serum albumin for 1 h and incubated with primary antibodies overnight at 4°C. GAPDH was used as an internal control. After washing with phosphate‐buffered saline (PBS), the membranes were incubated with the corresponding secondary antibodies at room temperature. After that, the membranes were incubated in Western Lightning™ Chemiluminescence Reagent (PerkinElmer, USA) for 5 min and visualized by LabWorks™ imaging system. The grey value of the target band was analysed with ImageJ software (National Institutes of Health, Bethesda, MD).

### 2.15 Periodic acid‐Schiff (PAS) stain

Mice were perfused with PBS, and then the renal tissues were fixed in 10% neutral buffered formalin overnight and dehydrated through graded alcohol series prior to processing for histology. The renal tissues were embedded in paraffin and stained with PAS reagent.

### 2.16 Transmission electron microscopy

Renal tissues were quickly cut into small pieces and fixed in 2.5% glutaraldehyde overnight. After embedding in epoxy resin, the ultrathin tissue sections were stained with uranyl acetate and lead citrate, and then examined with transmission electron microscope (HITACHI, Japan) and photographed.

### 2.17 Statistical analysis

All data were expressed as mean ± SD. SPSS version 18.0 (SPSS, Chicago, IL) was performed for statistical analysis. The statistical difference between the two groups was measured using Student's *t*‐test. Multiple groups were compared using a one‐way analysis of variance (ANOVA). The *p* < 0.05 was considered to be statistically significant.

## 3 RESULTS

### 3.1 LncRNA Glis2 was down‐expressed in high‐glucose cultured mouse podocytes

**FIGURE 1:**
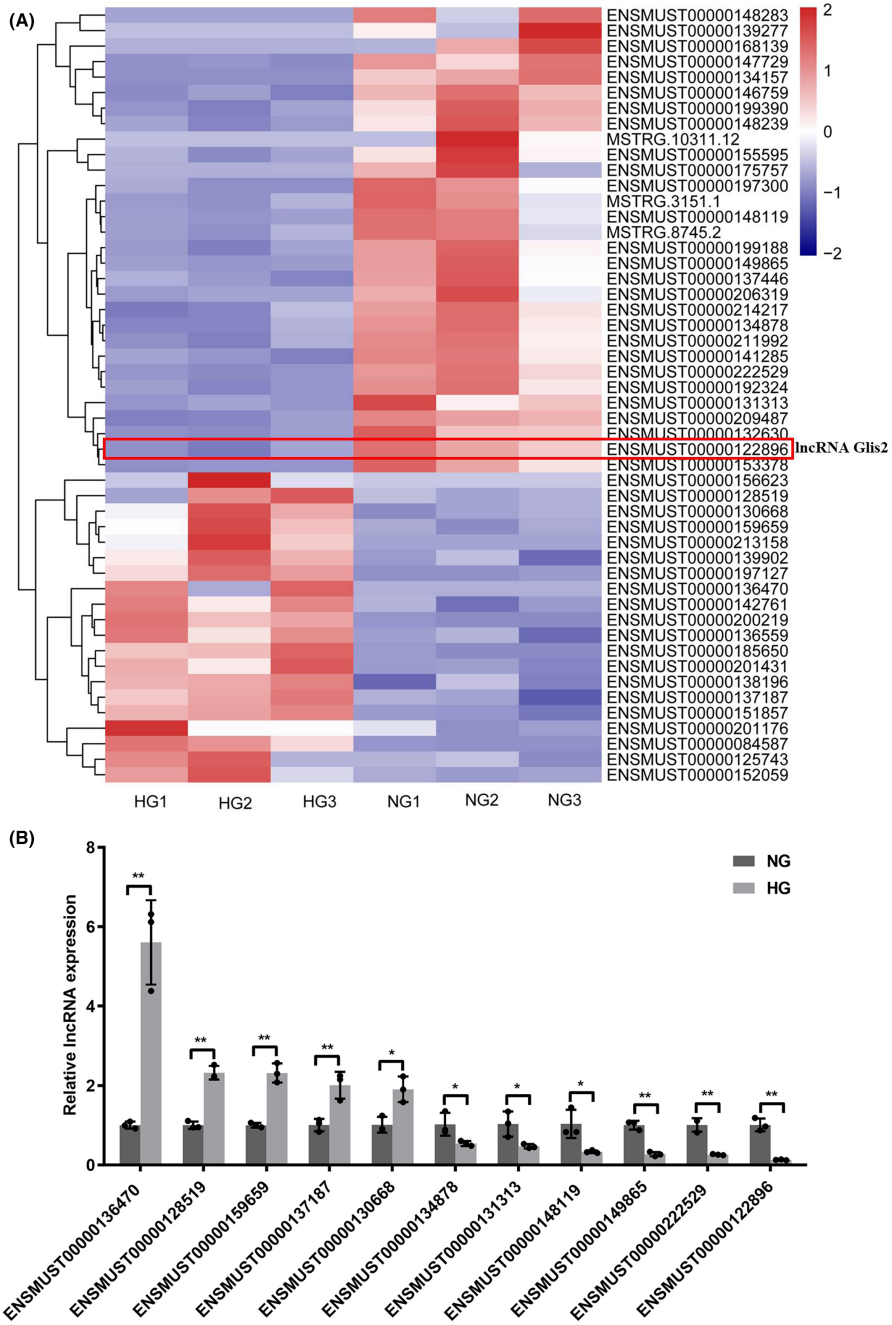
(A) RNA‐seq analysis showed that 51 lncRNAs were differentially expressed in podocytes between NG and HG groups. (B) Eleven lncRNAs were further validated by qRT‐PCR and were in consistent with RNA‐Seq analysis. The expression of lncRNA Glis2 was down‐expressed in high‐glucose cultured podocytes. Data were presented as mean ± SD. (*p < 0.05; **p < 0.01; ns, no significant).

To explore the effect of hyperglycemia on podocytes, mouse podocytes were cultured under normal‐glucose or high‐glucose concentration for 48 h. We confirmed that hyperglycemia could induce podocyte apoptosis (Figure S1A–E). In our study, RNA‐seq analysis showed that a total of 51 lncRNAs were differentially expressed between NG and HG groups using the following criteria: *p* value <0.001, *q* value <0.01 and |log2 (fold change)| > 1, including 20 up‐regulated and 31 down‐regulated genes (Figure 1A). Among them, the expressions of 11 lncRNAs were successfully validated using qRT‐PCR in NG and HG‐cultured podocytes. Data showed that the expressions of these candidate lncRNAs were consistent with the result of RNA‐seq (Figure 1B). Among these, the expression of ENSMUST00000122896 (lncRNA Glis2 for short) was markedly altered in the HG group compared with that in the NG group. There was about a 7.76‐fold change reduction of lncRNA Glis2 in the HG group compared with that in the NG group. Therefore, lncRNA Glis2 was focused on for further study to reveal the potential function and mechanism.

### 3.2 LncRNA Glis2 overexpression alleviated podocyte apoptosis and mitochondrial dysfunction induced by hyperglycemia

**FIGURE 2:**
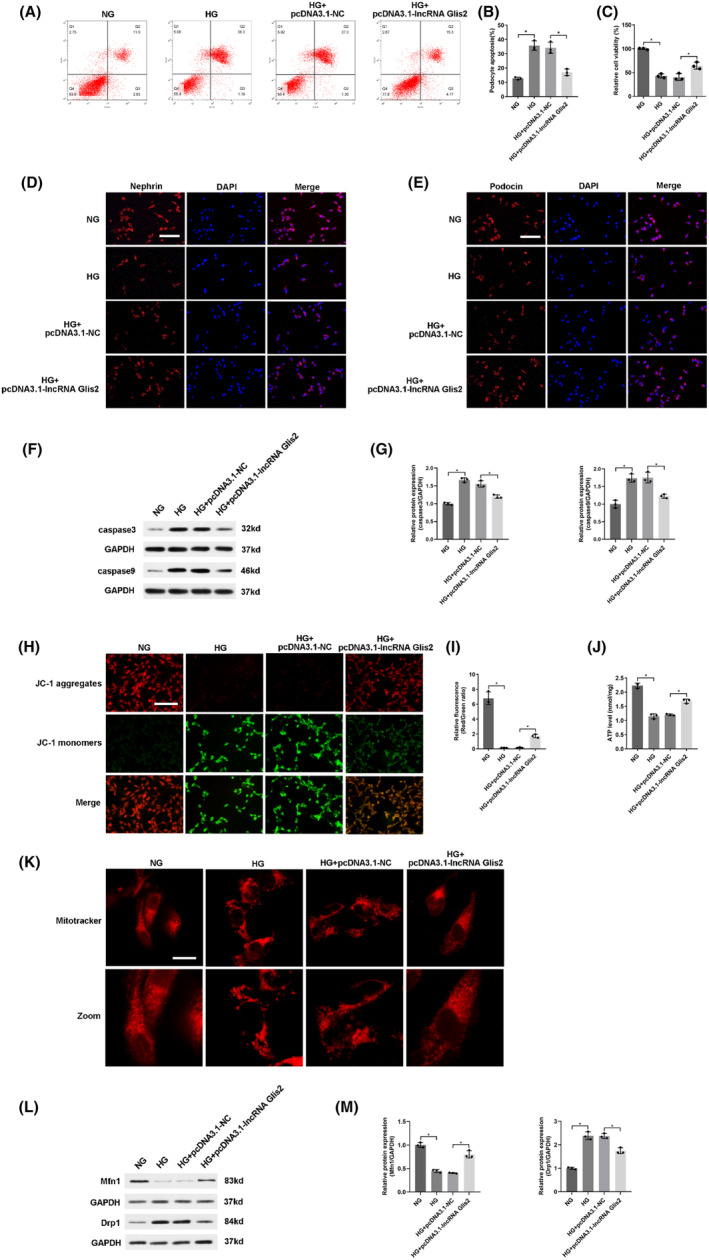
LncRNA Glis2 overexpression alleviated high‐glucose‐induced podocyte apoptosis and mitochondrial dysfunction. (A) Flow cytometry was performed to analyse the effect of lncRNA Glis2 overexpression on podocyte apoptosis under high‐glucose condition. (B) Quantitative analysis of apoptosis rate. (C) Quantitative analysis of relative cell viability. (D‐E) Representative images showed the expressions of nephrin and podocin by immunofluorescence. Scale bar: 100 μm. (F) The protein expressions of apoptosis‐related factors (caspase3 and caspase9) were detected by western blot in high‐glucose cultured podocytes transfected with pcDNA3.1‐lncRNA Glis2. GAPDH was utilized as internal reference. (G) Quantitative analysis of caspase3 and caspase9. (H) The ΔΨM was detected by JC‐1 staining in high‐glucose cultured podocytes transfected with pcDNA3.1‐lncRNA Glis2. Scale bar: 100 μm. (I) Quantitative analysis of ΔΨM. (J) ATP concentrations were measured by ELASA. (K) Representative micrographs showed mitochondrial morphology by MitoTracker Deep Red staining. Scale bar: 10 μm. (L) The protein levels of Mfn1 and Drp1 were determined by western blot. GAPDH was utilized as internal reference. (M) Quantitative analysis of Mfn1 and Drp1. Data were presented as mean ± SD (*n* = 3). *p < 0.05; ns, no significant.

To research the functional role of lncRNA Glis2 in podocyte apoptosis, we first successfully used pcDNA3.1 vector or chemically modified siRNA to transfect into normal‐glucose cultured podocytes. The results indicated that lncRNA Glis2 down‐regulation induced by hyperglycemia might be associated with podocyte apoptosis (Figure S2A–G). To further confirm whether lncRNA Glis2 overexpression alleviated podocyte apoptosis induced by hyperglycemia, pcDNA3.1‐lncRNA Glis2 was used to transfect into high‐glucose cultured podocytes. Flow cytometry showed that lncRNA Glis2 overexpression could alleviate podocyte apoptosis induced by hyperglycemia (Figure 2A,B). CCK‐8 assay revealed that lncRNA Glis2 overexpression could increase cell viability in HG + pcDNA3.1‐lncRNA Glis2 group (Figure 2C). Immunofluorescence assay revealed that the down‐regulation of nephrin and podocin induced by hyperglycemia was significantly reversed by transfected with pcDNA3.1‐lncRNA Glis2 (Figure 2D,E). In addition, lncRNA Glis2 overexpression significantly reversed the protein expressions associated with apoptosis in HG + pcDNA3.1‐lncRNA Glis2 group, including decreasing caspase3 and caspase9 (Figure 2F,G). Therefore, these data indicated that lncRNA Glis2 overexpression played a significant role in alleviating podocyte apoptosis induced by hyperglycemia.

Podocytes, which are terminally differentiated visceral epithelial cells, have a limited capacity for renewal and cannot regenerate when they suffer from damage. Thus, podocytes rely on their ability to maintain their complex structure via regulation and organization of the actin cytoskeleton and extracellular matrix. This imposes a high energy requirement and requires large amounts of mitochondria to supply energy.^9^, ^10^ To explore whether lncRNA Glis2 is involved in podocyte mitochondrial function, we first successfully used pcDNA3.1 vector or chemically modified siRNA to transfect into normal‐glucose cultured podocytes. The results indicated that lncRNA Glis2 might be associated with mitochondrial dysfunction in podocytes (Figure S2H–M). Next, we assessed whether lncRNA Glis2 overexpression alleviated mitochondrial dysfunction in high‐glucose cultured podocytes. Hyperglycemia exposure caused a large accumulation of JC‐1 monomers, indicating the occurrence of decreased ΔΨM. A decline in the ΔΨm is a distinctive feature of change in mitochondrial activity during early‐stage apoptosis of podocytes. More accumulation of JC‐1 aggregates and less diffuse JC‐1 monomers were observed in HG + pcDNA3.1‐lncRNA Glis2 group, which indicated that the decrease of ΔΨM caused by hyperglycemia significantly reversed by lncRNA Glis2 overexpression (Figure 2H,I). ATP concentration was decreased in the HG group than NG group. LncRNA Glis2 overexpression could increase ATP concentration in the HG + pcDNA3.1‐lncRNA Glis2 group compared with the HG group (Figure 2J). The change in mitochondrial morphology was observed using MitoTracker Deep Red staining. Under high‐glucose condition, mitochondria appeared rod‐shaped in podocytes. Highly fragmented and discrete mitochondria were observed in the HG group. LncRNA Glis2 overexpression significantly prevented hyperglycemia‐induced changes in mitochondrial morphology (Figure 2K). Mitofusin 1 (Mfn1) was significantly decreased and dynamin‐related protein 1 (Drp1) was increased in the HG group compared with the NG group. The changes in mitochondrial fusion and fission protein induced by hyperglycemia were reversed in HG + pcDNA3.1‐lncRNA Glis2 group (Figure 2L,M). These results suggested that lncRNA Glis2 overexpression alleviated podocyte apoptosis induced by hyperglycemia by restoring mitochondrial dysfunction in podocytes.

### 3.3 LncRNA Glis2 played biological function by serving as a competing endogenous RNA (ceRNA) of miR‐328‐5p

**FIGURE 3:**
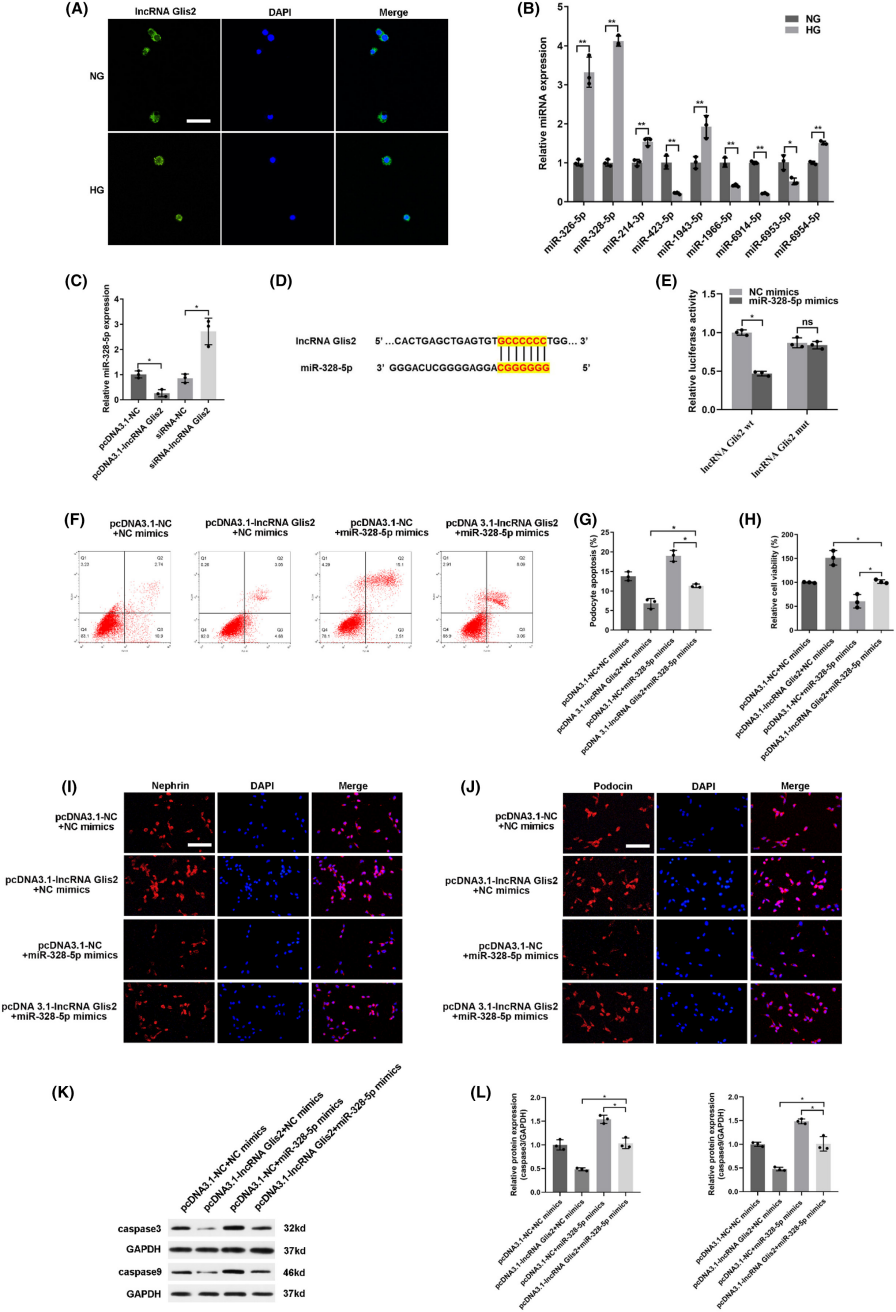
LncRNA Glis2 regulated podocyte apoptosis by directly targeting miR‐328‐5p. (A) The subcellular location of lncRNA Glis2 was detected by FISH. LncRNA Glis2 was mainly distributed in the cytoplasm of podocytes. (B) The potential miRNAs interacting with lncRNA Glis2 were measured by qRT‐PCR after transfecting with siRNA‐lncRNA Glis2. (C) The expression of miR‐328‐5p regulated by pcDNA3.1‐lncRNA Glis2 or siRNA‐lncRNA Glis2 was measured by qRT‐PCT. (D) The target binding site between lncRNA Glis2 and miR‐328‐5p was predicted by bioinformatics analysis. (E) Dual‐luciferase reporter assay was performed to measure the luciferase activity of co‐transfecting with miR‐328‐5p mimics or NC mimics and lncRNA Glis2 wt or mut luciferase reporters. Data were presented as the relative ratio of firefly luciferase activity to Renilla luciferase activity. (F) Flow cytometry was performed to analyse the effect of pcDNA3.1‐lncRNA Glis2 combined with miR‐328‐5p mimics on podocyte apoptosis. (G) Quantitative analysis of apoptosis rate. (H) Quantitative analysis of relative cell viability. (I‐J) Representative images showed the expressions of nephrin and podocin in podocytes co‐transfected with pcDNA3.1‐lncRNA Glis2 and miR‐328‐5p mimics. Scale bar: 100 μm. (K) Protein expression of apoptosis‐related factors (caspase3 and caspase9) regulated by the effect of pcDNA3.1‐lncRNA Glis2 combined with miR‐328‐5p mimics were measured by western blot. GAPDH was utilized as internal reference. (L) Quantitative analysis of caspase3 and caspase9. Data were presented as mean ± SD (*n* = 3). *p < 0.05; ns, no significant.

To further explore the potential mechanism of lncRNA Glis2 in podocyte apoptosis, we detected the subcellular location of lncRNA Glis2 by FISH. The results showed that lncRNA Glis2 was predominantly located in the cytoplasm of cells (Figure 3A). As known, if the lncRNAs are located in the cytoplasm, they mainly participate in post‐transcriptional gene regulation by acting as a ceRNA or forming complexes with specific protein. Thus, we speculated that lncRNA Glis2 could regulate the target genes by competitively binding miRNA as a ceRNA.

To further elucidate the mechanism of lncRNA Glis2 in podocyte apoptosis, we used a microarray chip and bioinformatics to interrogate the potential miRNAs interacting with lncRNA Glis2. Nine candidates of putative miRNA for lncRNA Glis2 were predicted online databases (StarBase, miRbase, USCS, and BiBiserv2 software). Among these, miR‐328‐5p was chosen as the predicted candidate because it was the highest after transfecting with siRNA‐lncRNA Glis2 (Figure 3B). Data from qRT‐PCR also revealed that the expression of miR‐328‐5p is negatively correlated with lncRNA Glis2 (Figure 3C). Figure 3D illustrates the binding sequence prediction of lncRNA Glis2 and miR‐328‐5p. To validate the bioinformatics results, the direct interaction between lncRNA Glis2 and miR‐328‐5p was confirmed by dual luciferase reporter assay (Figure 3E). We constructed wild‐type and mutant 3′‐UTR lncRNA Glis2 luciferase reporter vectors. A wild‐type plasmid lncRNA Glis2‐wt or mutant plasmid lncRNA Glis2‐mut were co‐transfected with miR‐328‐5p mimics or NC mimics in podocytes and the luciferase activity was examined. When lncRNA Glis2‐wt was used for co‐transfecting, the luciferase activity was striking inhibited in the miR‐328‐5p mimics group compared with the NC mimics group. Whereas, when lncRNA Glis2‐mut was used for co‐transfecting, there was no significant difference between miR‐328‐5p mimics and the NC mimics group. Taken together, these findings confirmed that there was a direct interaction between lncRNA Glis2 and miR‐328‐5p.

To further validate whether lncRNA Glis2 could interact with miR‐328‐5p to affect podocyte apoptosis, rescue experiments were performed by co‐transfecting pcDNA3.1‐lncRNA Glis2 and miR‐328‐5p mimics. As shown, up‐regulation of miR‐328‐5p could apparently induce podocyte apoptosis, but co‐transfection with pcDNA3.1‐lncRNA Glis2 could partially reverse the effect. The ability of inhibiting podocyte apoptosis which was caused by lncRNA Glis2 overexpression was partly countervailed by co‐transfection with miR‐328‐5p mimics (Figure 3F–L). Taken together, the above results elucidated that lncRNA Glis2 regulated podocyte apoptosis by interacting with miR‐328‐5p.

### 3.4 Inhibiting of miR‐328‐5p alleviated podocyte apoptosis and mitochondrial dysfunction

**FIGURE 4:**
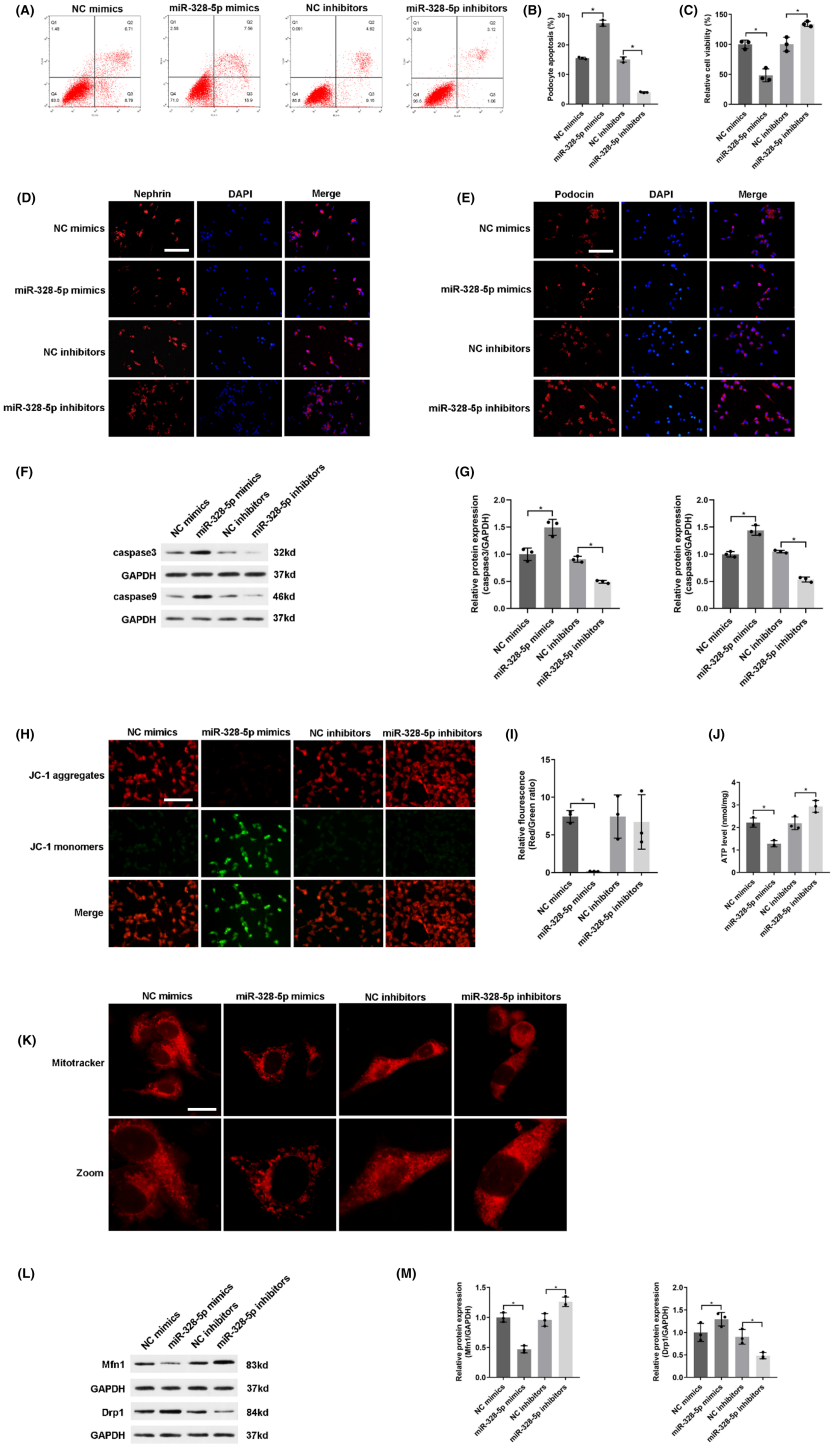
MiR‐328‐5p up‐regulation or down‐regulation regulated podocyte apoptosis and mitochondrial dysfunction in normal‐glucose cultured podocytes transfected with miR‐328‐5p mimics or inhibitor. (A) The apoptosis rate of podocytes was measured by flow cytometry. (B) Quantitative analysis of apoptosis rate. (C) Quantitative analysis of relative cell viability. (D‐E) The expressions of nephrin and podocin in podocytes were evaluated by immunofluorescence. Scale bar: 100 μm. (F) Western blot was used to analyse the protein expression of apoptosis‐related factors (caspase3 and caspase9). GAPDH was used as a loading control. (G) Quantitative analysis of caspase3 and caspase9. (H) The ΔΨM was detected by JC‐1 staining. Scale bar: 100 μm. (I) Quantitative analysis of ΔΨM. (J) ATP concentrations were determined by ELASA. (K) Representative micrographs showed mitochondrial morphology by MitoTracker Deep Red staining. Scale bar: 10 μm. (L) Representative bands of Mfn1 and Drp1 expressions were detected by western blot. GAPDH was used as a loading control. (M) Quantitative analysis of Mfn1 and Drp1. Data were presented as mean ± SD (*n* = 3). *p < 0.05; ns, no significant.

In order to investigate the function of miR‐328‐5p in podocyte apoptosis, we used miR‐328‐5p mimics or miR‐328‐5p inhibitors to transfect into a panel of normal‐glucose cultured podocytes. Flow cytometry revealed that miR‐328‐5p mimics significantly induced podocyte apoptosis, whereas miR‐328‐5p inhibitors ameliorated podocyte apoptosis (Figure 4A,B). Next, the results of the CCK‐8 assay showed that miR‐328‐5p mimics decreased cell viability, while inhibiting the expression of miR‐328‐5p increased cell viability (Figure 4C). Moreover, immunofluorescence assay revealed that miR‐328‐5p mimics obviously reduced the protein expression of nephrin and podocin, whereas miR‐328‐5p inhibitors up‐regulated the expression of nephrin and podocin (Figure 4D,E). Western blot displayed that pro‐apoptotic caspase3 and caspase9 were significantly increased in the miR‐328‐5p mimics group. On the other hand, the expressions of caspase3 and caspase9 were reversed respectively once miR‐328‐5p was knock down in the miR‐328‐5p inhibitors group (Figure 4F,G). Therefore, these results confirmed that up‐regulation of miR‐328‐5p induced podocyte apoptosis, whereas inhibition of miR‐328‐5p alleviated podocyte apoptosis.

Furthermore, we assessed the role of miR‐328‐5p in mitochondrial dysfunction. In contrast to the NC mimics group, overexpression of miR‐328‐5p caused a large accumulation of JC‐1 monomers, indicating the decline of ΔΨM in miR‐328‐5p mimics group (Figure 4H,I). Overexpression of miR‐328‐5p markedly reduced ATP concentration in the miR‐328‐5p mimics group than the NC mimics group (Figure 4J). Meanwhile, overexpression of miR‐328‐5p significantly affected mitochondrial morphology, including more fragmented and discrete mitochondria (Figure 4K). Western blot displayed that up‐regulation of miR‐328‐5p decreased the expression of Mfn1 and increased the expression of Drp1 (Figure 4L,M). However, inhibition of miR‐328‐5p had an adverse effect on mitochondrial function. These data suggested that overexpression of miR‐328‐5p induced mitochondrial dysfunction, while inhibition of miR‐328‐5p alleviated mitochondrial dysfunction.

### 3.5 MiR‐328‐5p targets Sirt1 in podocyte apoptosis

**FIGURE 5:**
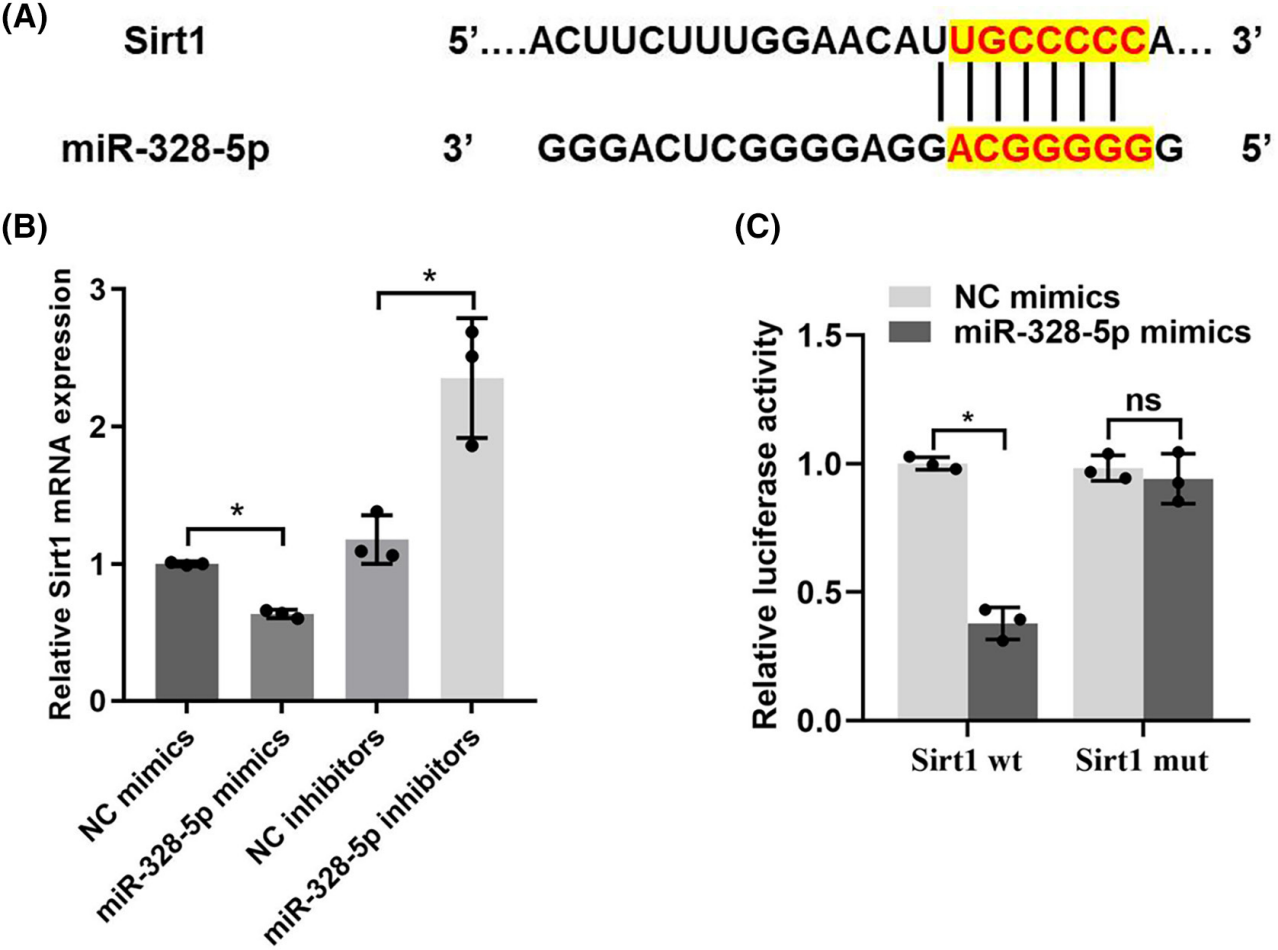
(A) The predicted target binding site between miR‐328‐5p and Sirt1 was shown. (B) The expression of Sirt1 regulated by miR‐328‐5p mimics or inhibitors was measured by qRT‐PCT. (C) Dual‐luciferase reporter assay was performed to measure the luciferase activity of co‐transfecting with miR‐328‐5p mimics or NC mimics and Sirt1 wt or mut luciferase reporters. Data were presented as the relative ratio of firefly luciferase activity to Renilla luciferase activity. *p < 0.05; ns, no significant.

In order to further decipher the mechanism of miR‐328‐5p in podocyte apoptosis, we predicted the potential target genes that miR‐328‐5p may regulate by bioinformatics analysis (TargetScan, http://www.targetscan.org). Among these, Sirt1 was chosen as the predicted candidate because it was reported to be related to mitochondrial function and podocyte apoptosis.^11^, ^12^, ^13^ We found that there was a direct binding site between miR‐328‐5p and Sirt1 (Figure 5A). In addition, up‐regulation of miR‐328‐5p reduced the expression of Sirt1. However, the expression of Sirt1 was increased when miR‐328‐5p was inhibited (Figure 5B). Importantly, dual luciferase reporter assay revealed that miR‐328‐5p mimics co‐transfection with a wild‐type Sirt1 reporter gene (Sirt1‐wt) could reduce the relative luciferase activities. Nevertheless, the relative luciferase activities were not significantly changed when miR‐328‐5p mimics were co‐transfected with a mutant Sirt1 reporter gene (Sirt1‐mut) (Figure 5C). These data indicated that miR‐328‐5p negatively regulated the expression of Sirt1 in a direct manner.

### 3.6 Sirt1 overexpression alleviated podocyte apoptosis and mitochondrial dysfunction induced by hyperglycemia

**FIGURE 6:**
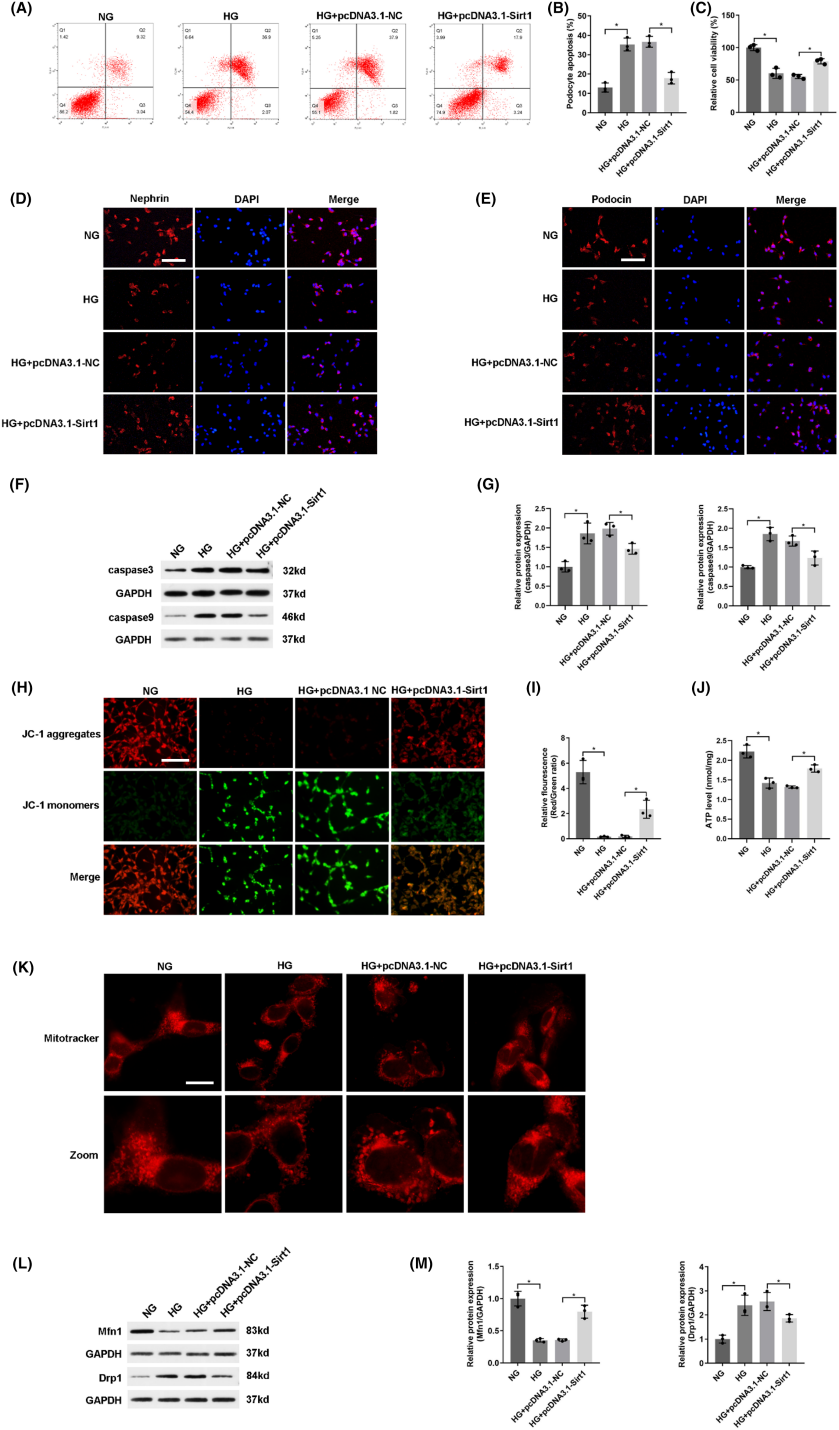
Up‐regulation of Sirt1 alleviated podocyte apoptosis and mitochondrial dysfunction induced by hyperglycemia. (A) Podocyte apoptosis was measured by flow cytometry in high‐glucose cultured podocytes transfected with pcDNA3.1‐Sirt1. (B) Quantitative analysis of apoptosis rate. (C) Quantitative analysis of relative cell viability. (D‐E) Immunofluorescence staining revealed the expressions of nephrin and podocin. Scale bar: 100 μm. (F) The protein expressions of apoptosis‐related factors (caspase3 and caspase9) were shown. (G) Quantitative analysis of caspase3 and caspase9. (H) The ΔΨM was measured by JC‐1 staining in high‐glucose cultured podocytes transfected with pcDNA3.1‐Sirt1. Scale bar: 100 μm. (I) Quantitative analysis of ΔΨM. (J) ATP concentrations were measured by ELASA. (K) Representative micrographs showed mitochondrial morphology by MitoTracker Deep Red staining. Scale bar: 10 μm. (L) The protein expressions of Mfn1 and Drp1 were detected by western blot. GAPDH was utilized as internal reference. (M) Quantitative analysis of Mfn1 and Drp1. Data were presented as mean ± SD (*n* = 3). *p < 0.05; ns, no significant.

To identify the biological function of Sirt1, we investigated the effect of Sirt1 overexpression by transfecting pcDNA3.1‐Sirt1 in podocyte apoptosis induced by hyperglycemia. Strikingly, Sirt1 overexpression reduced podocyte apoptosis (Figure 6A,B) and increased cell viability (Figure 6C). Sirt1 overexpression significantly prevented the down‐regulation of nephrin and podocin induced by hyperglycemia (Figure 6D,E). Up‐regulation of Sirt1 significantly reversed the protein expressions associated with apoptosis, including decreasing caspase3 and caspase9 (Figure 6F,G). Therefore, these results confirmed that up‐regulation of Sirt1 alleviated podocyte apoptosis induced by hyperglycemia.

To further evaluate whether Sirt1 overexpression alleviated mitochondrial dysfunction induced by hyperglycemia, pcDNA3.1‐Sirt1 was transfected into high‐glucose cultured podocytes. The decrease of ΔΨM (Figure 6H,I), the down‐regulation of ATP (Figure 6J) and abnormal mitochondrial morphology (Figure 6K) caused by hyperglycemia were significantly reversed by transfection with pcDNA3.1‐Sirt1. Furthermore, western blot showed that these abnormal changes in mitochondrial fusion or fission protein induced by hyperglycemia were impeded by Sirt1 overexpression (Figure 6L,M). Collectively, these results elucidated that up‐regulation of Sirt1 alleviated mitochondrial dysfunction induced by hyperglycemia in podocytes.

### 3.7 Up‐regulation of lncRNA Glis2 attenuated podocyte apoptosis in diabetic mice

**FIGURE 7:**
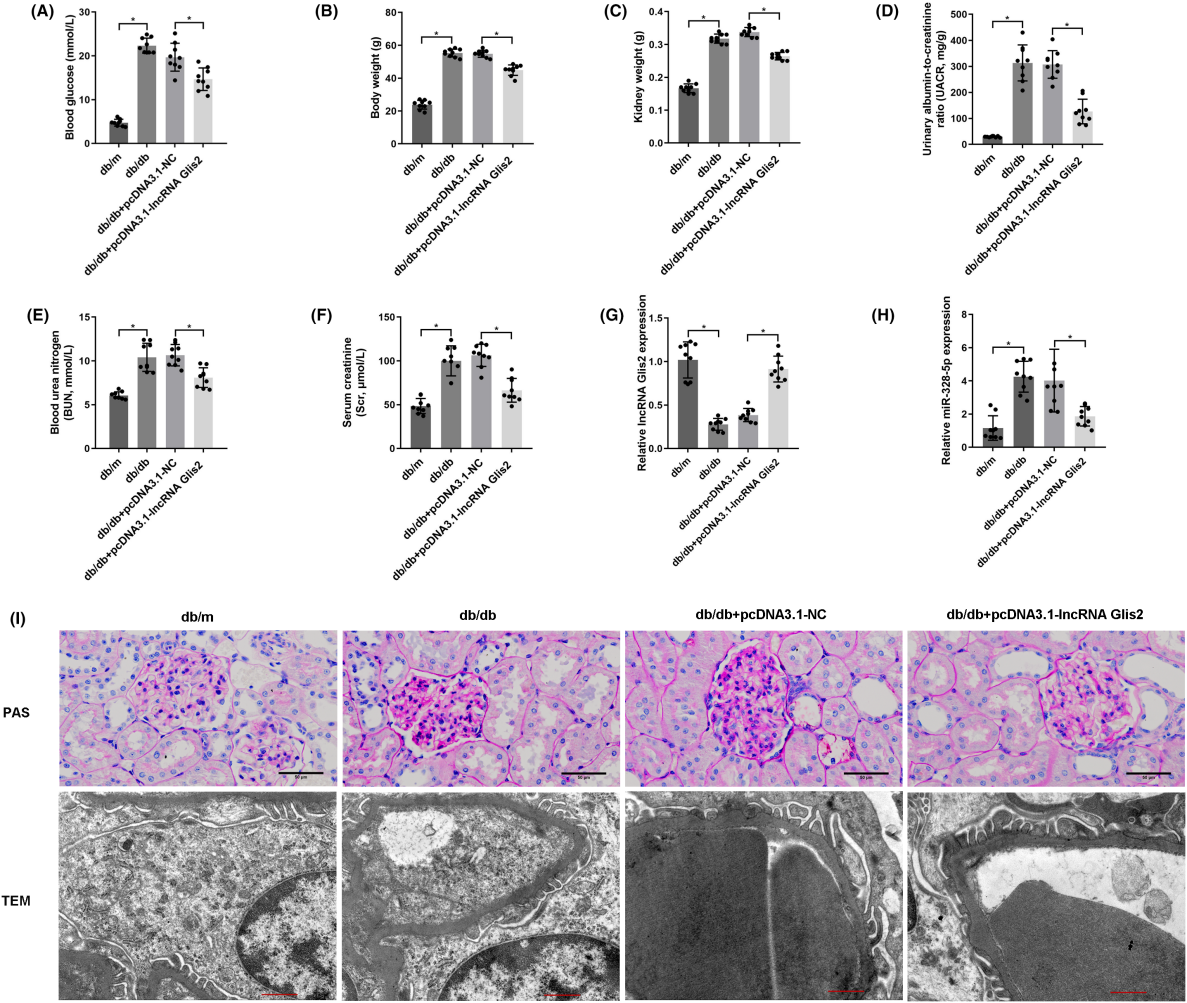
LncRNA Glis2 overexpression lentivirus attenuated podocyte apoptosis in db/db mice. (A‐F) Blood glucose (mmol/L), body weight (g), kidney weight (g), urinary albumin‐to‐creatinine ratio (mg/g), blood urea nitrogen (mmol/L) and serum creatinine (μmol/L) were detected. (G‐H) The expression of lncRNA Glis2 and miR‐328‐5p in kidney tissue was measured by qRT‐PCR. (I) Representative images of paraffin‐embedded sections stained with Periodic Acid‐Schiff (PAS) are shown. Scale bar: 50 μm. Transmission electron microscopy (TEM) assay showed the ultrastructure of the podocytes in renal cortex of db/db mice. Scale bar: 1.0 μm (*n* = 3. *p < 0.05; ns, no significant).

The above findings demonstrated that up‐regulation of lncRNA Glis2 might be a valuable therapeutic approach in DN. To verify whether lncRNA Glis2 also attenuated podocyte apoptosis in the same way in diabetic mice, lncRNA Glis2 overexpression lentivirus was transfected into db/db mice by tail vein injection. Compared with those in db/db + pcDNA3.1‐NC group, analyses of biochemical parameters (Figure 7A–F), including blood glucose, body weight, kidney weight, UACR, BUN and Scr, were evidently lower in db/db + pcDNA3.1‐lncRNA Glis2 group. Compared with that in db/db + pcDNA3.1‐NC group, a significant increase in the expression of lncRNA Glis2 (Figure 7G) and a significant decrease in the expression of miR‐328‐5p (Figure 7H) in kidney tissue were detected in db/db + pcDNA3.1‐lncRNA Glis2 group.

PAS staining revealed the stromal hyperplasia and thickened glomerular basement membrane in db/db mice. LncRNA Glis2 overexpression lentivirus treatment produced dramatic reductions in these pathological changes in the db/db + pcDNA3.1‐lncRNA Glis2 group. TEM confirmed the alleviation of podocyte foot process effacement and glomerular basement membrane thickening in the db/db + pcDNA3.1‐lncRNA Glis2 group compared with the db/db + pcDNA3.1‐NC group (Figure 7I).

## 4 DISCUSSION

Our findings first demonstrated that unidentified lncRNA Glis2 was markedly down‐regulated and associated with podocyte apoptosis in high‐glucose cultured podocytes and diabetic db/db mice. More importantly, lncRNA Glis2 overexpression alleviated podocyte apoptosis by reducing mitochondrial dysfunction. Further, lncRNA Glis2 exerted biological function by serving as a ceRNA of miR‐328‐5p, which negatively regulated the expression of its target Sirt1 and downstream proteins. Overall, lncRNA Glis2, acting as a ceRNA of miR‐328‐5p, regulated Sirt1‐mediated mitochondrial function in podocyte apoptosis in DN. Targeting lncRNA Glis2 may be a therapeutic approach for podocyte apoptosis in DN.

The rising and emerging evidence has demonstrated that lncRNA effectively participated in the pathophysiological process of DN, such as LINC01619^8^ and lncRNA SPAG5‐AS1.^14^ In this study, we found that the expression of lncRNA Glis2 was down‐regulated in high‐glucose cultured podocytes. Overexpression of lncRNA Glis2 rescued podocyte apoptosis in podocytes under high‐glucose condition. Furthermore, lncRNA Glis2 overexpression attenuated podocyte apoptosis in diabetic mice. These results provided evidence that lncRNA Glis2 down‐regulation induced by hyperglycemia was an important reason for podocyte apoptosis. LncRNA Glis2 played a renoprotection role in DN.

Mitochondria are highly plastic organelles that constantly change their number, shape and intracellular localization through fusion and fission processes in response to energy demands.^15^ Mitochondrial dynamics is governed by the balance between these two opposite processes of fusion and fission.^16^ Mitochondrial fusion promotes the generation of interconnected mitochondria and is regulated by fusion proteins (mitofusins 1 and 2 (Mfn1, Mfn2) and optical atrophy (OPA1)). On the contrary, mitochondrial fission produces numerous mitochondrial fragments in order to remove the impaired mitochondria and is regulated by fission proteins (dynamin‐related protein 1 (Drp1) and mitochondrial fission 1 (Fis1)).^17^ Mitochondrial dysfunction leads to impaired ATP generation, increases oxidative stress and alters mitochondrial membrane permeability, resulting in cell dysfunction or cell apoptosis.^18^ Mitochondria are a major source of intracellular reactive oxygen species (ROS) generation in the majority of cell types. ROS can increase the release of mitochondrial intermembrane space proteins to the cytoplasm,^19^ which leads to activation of the caspase cascade (apoptotic markers) and mitochondria‐mediated apoptosis.^20^ Accumulating studies have indicated that improving mitochondrial function attenuated high‐glucose‐induced podocyte apoptosis.^21^, ^22^ Consistent with this, the current study revealed that overexpression of lncRNA Glis2 attenuated mitochondrial dysfunction in podocytes under high‐glucose condition.

LncRNA Glis2 (Ensembl ID: ENSMUST00000122896), which is a 3512 bp lncRNA, is located in chromosome 16 (Chr16: 4426491–4433806). A large amount of evidence has demonstrated that lncRNAs could participate in diabetes and diabetic complications by acting as ceRNA.^23^, ^24^ In this study, we speculated that lncRNA Glis2 may have participated in podocyte mitochondria dysfunction by acting as a ceRNA. We predicated the target gene of lncRNA Glis2 by bioinformatics analysis and found that miR‐328‐5p is its potential binding target. QRT‐PCR data showed that the expression of miR‐328‐5p was opposite to lncRNA Glis2. The direct relationship between lncRNA Glis2 and miR‐328‐5p was further confirmed using a dual luciferase reporter gene assay. These results indicated that lncRNA Glis2 participate in podocyte apoptosis maybe through directly binding to miR‐328‐5p.

Additionally, the role of miRNAs in mitochondrial function has been elucidated in previous research. A previous study showed that miR‐328 regulated cell resistance to complement‐dependent cytotoxicity (CDC) by modifying the expression of membrane complement regulators CD46 and CD59 and the response of the mitochondria to complement lytic attack.^25^ In the current study, a negative feedback regulatory mechanism between lncRNA Glis2 and miR‐328‐5p was demonstrated. Furthermore, miR‐328‐5p overexpression aggravated mitochondrial dysfunction and podocyte apoptosis.

Subsequently, this study confirmed that miR‐328‐5p effectively inhibited Sirt1 expression by directly binding to the 3′UTRs of its mRNAs. The Sirt1 expression and its effect on podocyte apoptosis was opposite to miR‐328‐5p. The direct relationship between miR‐328‐5p and Sirt1 was also verified using a dual luciferase reporter assay. Both podocyte‐specific Sirt1 overexpression and Sirt1 agonist treatment could attenuate diabetes‐induced podocyte apoptosis and effectively alleviate the progression of diabetic kidney disease in diabetic mice.^12^ Consistent with previous studies, this present study reconfirmed the protective effect of Sirt1 in mitochondrial function and podocyte apoptosis in DN. Based on the above findings, this study demonstrated that miR‐328‐5p regulated mitochondrial function and podocyte apoptosis by directly targeting Sirt1.

In summary, these results together revealed that lncRNA Glis2 overexpression ameliorated Sirt1‐mediated mitochondrial dysfunction and podocyte apoptosis by severing as a ceRNA of miR‐328‐5p.

## 5 CONCLUSION

**FIGURE 8:**
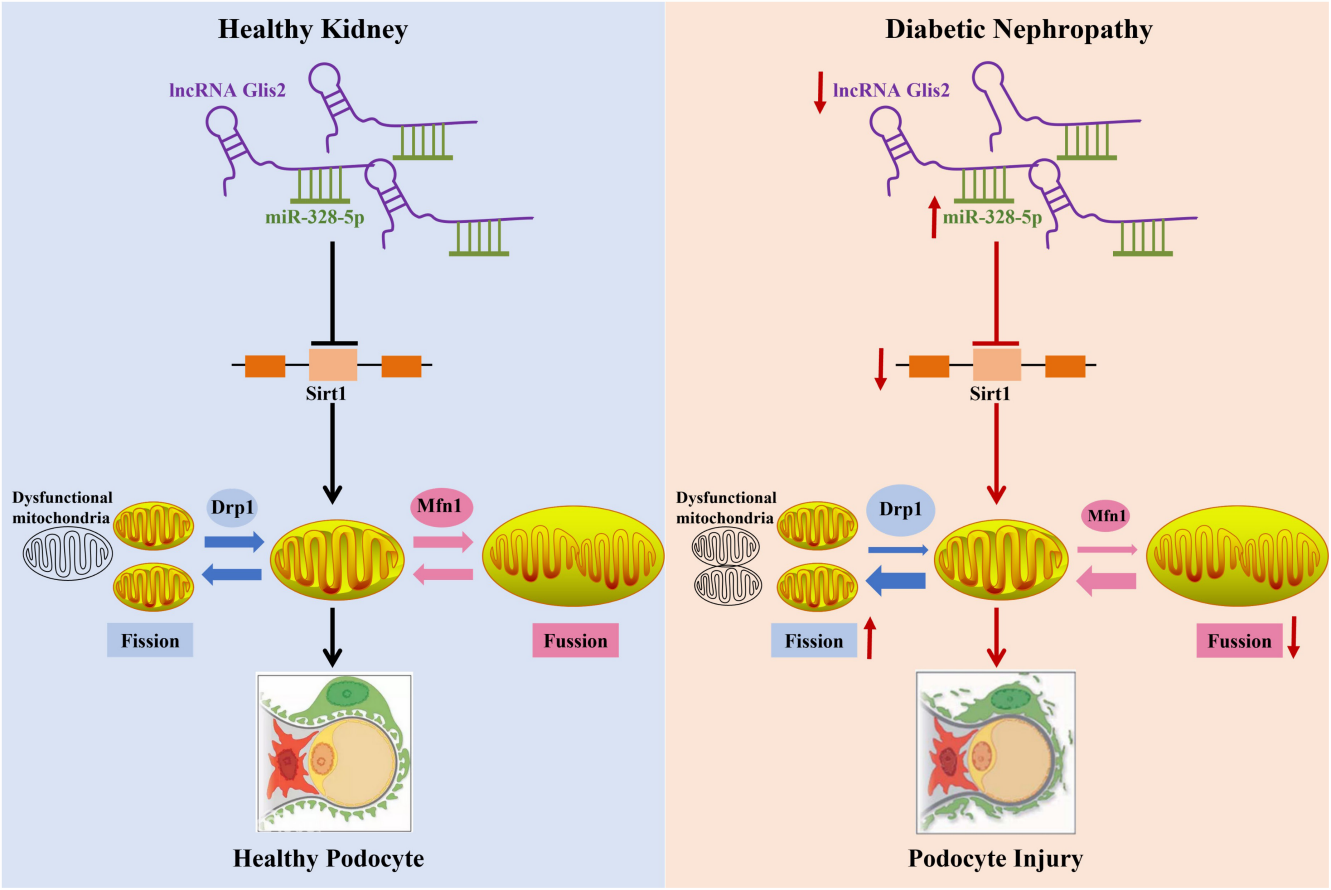
Mechanistic depiction of the role of lncRNA Glis2 in mitochondrial dysfunction and podocyte apoptosis was shown. Our study revealed that lncRNA Glis2 down‐regulation enhanced the inhibitory effect of miR‐328‐5p on Sirt1, resulting in mitochondrial dysfunction and podocyte apoptosis, which led to the progression of DN.

Our investigation unravelled that lncRNA Glis2 down‐regulation is an important reason for podocyte mitochondrial dysfunction and apoptosis in DN. LncRNA Glis2 down‐regulation promoted the inhibitory effect of miR‐328‐5p on Sirt1, thus triggering mitochondrial dysfunction and podocyte apoptosis in DN (Figure 8). This study provided new evidence into the mechanisms of renoprotection associated with lncRNA Glis2. The interaction between lncRNA Glis2 and miR‐328‐5p may provide a novel and specific therapeutic target for tackling podocyte apoptosis in DN. However, this current study had some limitations. We have only investigated the expression and molecular mechanism of lncRNA Glis2 in podocyte apoptosis, but we don't know how it is expressed in other renal cells. Whether the findings obtained in podocytes are consistent with other renal cell types needs to be further investigated. We intend to explore the expression and underlying molecular mechanism of lncRNA Glis2 in glomerular mesangial cells or other renal cell types in the near future.

## AUTHOR CONTRIBUTIONS

**Ting Wang:** Data curation (lead); formal analysis (lead); writing – original draft (lead). **Yanxia Chen:** Conceptualization (lead); funding acquisition (lead); supervision (lead); writing – review and editing (lead). **Zhihong Liu:** Investigation (equal); writing – review and editing (equal). **Jing Zhou:** Investigation (supporting); writing – review and editing (equal). **Na Li:** Data curation (supporting). **Yue Shan:** Data curation (equal); resources (equal). **Yinxi He:** Resources (supporting); software (supporting).

## FUNDING INFORMATION

This study was funded by the Natural Science Funds for Young Scholars of Hebei, China (Grant number: H2020206108), and the subject of the Health Commission of Hebei, China (Grant number: 20210151).

## CONFLICT OF INTEREST STATEMENT

The authors declare no competing interest.

## Supporting information


Figure S1.

Figure S2.

## Data Availability

All data and materials supporting the conclusions of this article were available in the figures, tables, and supplementary materials, which are available to authorized users.
