# Supplementary figures and images for: Long noncoding RNA Glis2 regulates podocyte mitochondrial dysfunction and apoptosis in diabetic nephropathy via sponging miR‐328‐5p
Source: J Cell Mol Med. 2024 Mar 20;28(7):e18204. doi: 10.1111/jcmm.18204 (PMC10951868; doi:10.1111/jcmm.18204)

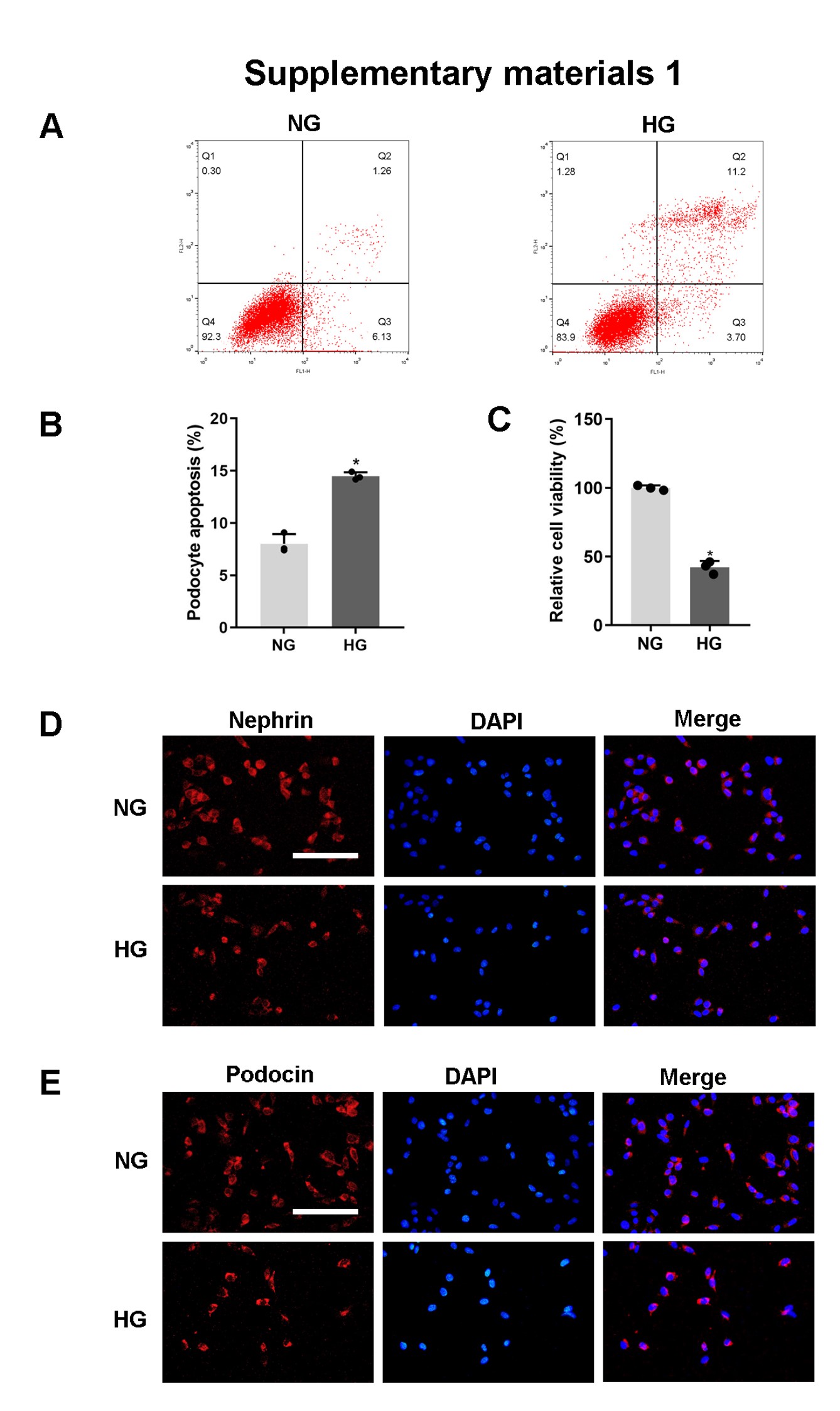

Supplement: Supplementary file 1 — Figure S1. [file JCMM-28-e18204-s002.jpg]

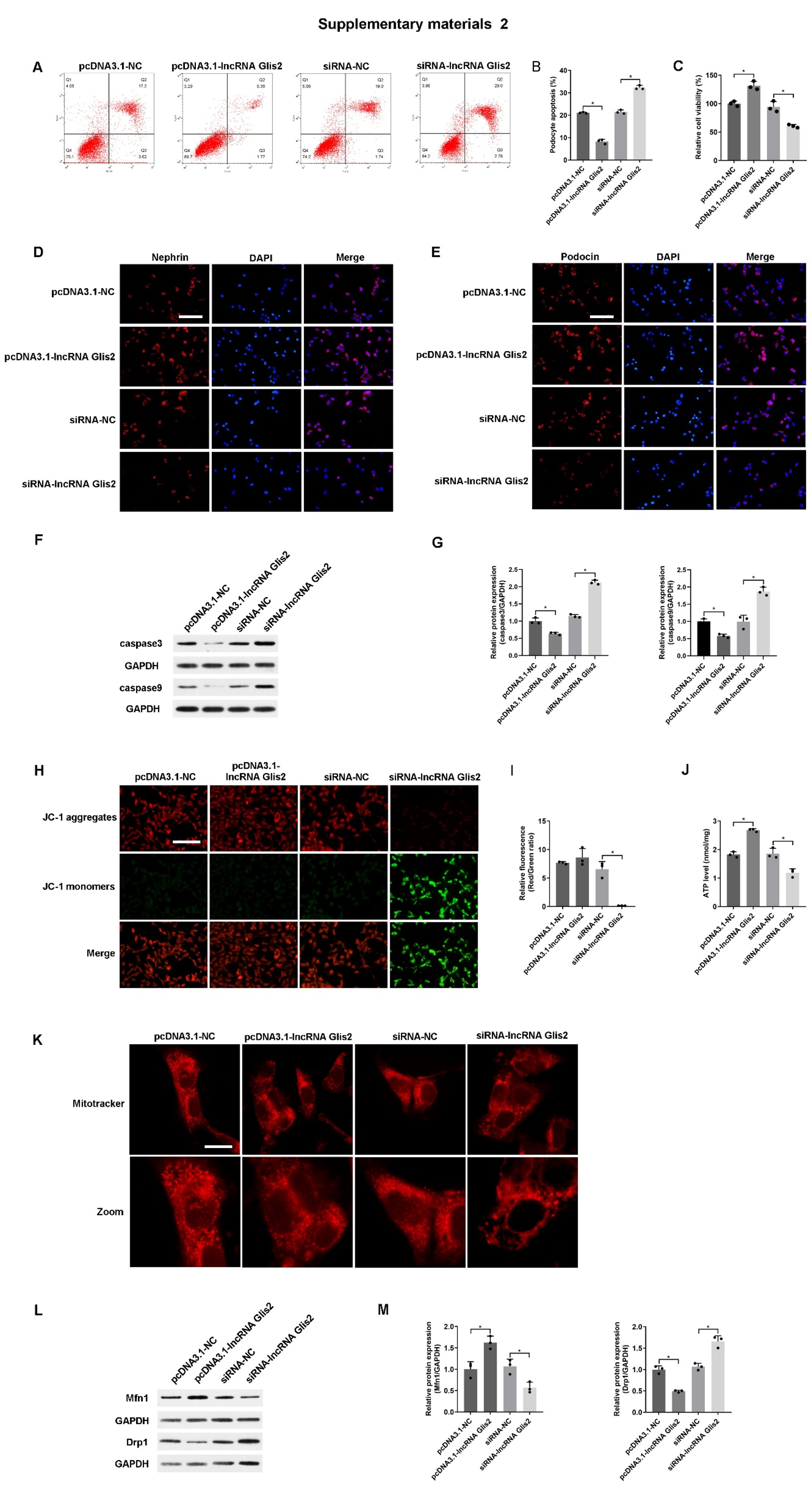

Supplement: Supplementary file 2 — Figure S2. [file JCMM-28-e18204-s001.jpg]
